# BTBD19 promotes colorectal cancer progression and correlates with adverse clinical outcomes

**DOI:** 10.3389/fonc.2025.1685601

**Published:** 2025-11-10

**Authors:** Changjiang Yang, Xuhua Geng, Zihan Zhao

**Affiliations:** 1 Aerospace Center Hospital, Peking University Aerospace School of Clinical Medicine, Beijing, China; 2 Department of Gastroenterological Surgery, Peking University People’s Hospital, Beijing, China; 3 School of Public Health, Boston University, Boston, MA, United States

**Keywords:** BTBD19, colorectal cancer, immune infiltration, prognosis, metastasis, immune checkpoints

## Abstract

**Background:**

Colorectal cancer (CRC) is a leading cause of cancer-related mortality worldwide. The identification of novel prognostic biomarkers and therapeutic targets is crucial for improving clinical management and patient outcomes. Members of the BTBD (BTB/POZ domain-containing) protein family have been implicated in tumorigenesis, but the role of BTBD19 in CRC remains poorly understood.

**Objective:**

This study aimed to investigate the expression pattern of BTBD19 in CRC, its association with clinicopathological features and prognosis, and its potential molecular mechanisms involving functional pathways and immune infiltration.

**Methods:**

BTBD19 expression was analyzed using public datasets (TCGA, GEO) and clinical tissue microarrays. Immunohistochemistry (IHC) was performed to validate protein expression. Survival analysis (OS, DSS, PFI) was conducted to assess prognostic significance. Functional enrichment analyses (GO/KEGG/GSEA) and immune infiltration analyses (ESTIMATE, ssGSEA, CIBERSORT) were used to explore underlying molecular mechanisms and immune-related associations.

**Results:**

BTBD19 was significantly upregulated in CRC tissues at both mRNA and protein levels compared to normal tissues. High BTBD19 expression was associated with advanced pathologic stages and poor prognosis (OS, DSS, PFI; all p<0.05). Functional analyses revealed that BTBD19-associated genes were enriched in pathways related to extracellular matrix organization, focal adhesion, and epithelial-mesenchymal transition. Immune infiltration analysis showed positive correlations between BTBD19 expression and stromal/immune scores, M2 macrophage infiltration, and expression of immune checkpoints (CD274, PDCD1).

**Conclusion:**

BTBD19 is upregulated in CRC and promotes tumor progression. It may serve as a potential prognostic biomarker for CRC, with implications for understanding CRC pathogenesis and immune microenvironment regulation.

## 1 Introduction

Colorectal cancer (CRC), ranked among the most common malignant tumors globally, poses a significant threat to human health (1). Although considerable advancements have been achieved in diagnostic and therapeutic approaches, treatment outcomes for a substantial portion of CRC patients still remain suboptimal (2, 3). The discovery of novel biomarkers and clarification of the underlying molecular mechanisms driving CRC progression are pivotal for enhancing clinical management and improving patient prognosis (4, 5).

The BTBD (BTB/POZ domain-containing) protein family, characterized by a conserved BTB domain, regulates diverse cellular processes, including protein ubiquitination, transcription, and cell signaling (6). Growing emerging evidence links members of the BTBD family to cancer progression. For instance, BTBD7 plays a role in the development of multiple tumors (7); BTBD3 inhibits colorectal cancer tumorigenesis by regulating the TYRO3/Wnt/β-catenin signaling axis; MiR-200b-5p suppresses tumor progression in salivary adenoid cystic carcinoma by targeting BTBD1 (8); and BTBD10 inhibits glioma tumorigenesis by downregulating cyclin D1 and p-Akt9.

While several BTBD family members have been linked to colorectal carcinogenesis, the role of BTBD19 in CRC remains unstudied. Here, we characterize BTBD1 (9) expression in CRC, assess its associations with clinicopathological features and prognosis, and use multi-omics analyses to explore its functional roles in tumor progression and immune infiltration-with the goal of evaluating BTBD19 as a potential CRC biomarker.

## 2 Materials and methods

### 2.1 Acquisition and processing of data

RNA-seq datasets with corresponding clinical information from CRC tumors and adjacent normal tissue samples were acquired from The Cancer Genome Atlas (TCGA, https://cancergenome.nih.gov/), a leading public resource for cancer genomics research. Additionally, the GSE110224 dataset utilized in this study was extracted from the Gene Expression Omnibus (GEO, http://www.ncbi.nlm.nih.gov/geo), a broadly accessible functional genomics repository that facilitates MIAME-standard data submissions (10).

### 2.2 Patient samples and clinical specimens

Clinical Specimens of Cohort 1 supplied by Shanghai Outdo Biotech Company (Shanghai, China), the CRC tissue microarray (HColA160CS01) comprised 80 paired tumor and adjacent normal tissue samples. Ethical approval for the study protocols was granted by the company’s Ethics Committee under the approval ID: YB M-05-02. Clinical Specimens of Cohort 2 Supplied by Shanghai Zhuoli Biotech Company (Shanghai, China), the CRC tissue microarray (ZL-RecA961) comprised 48 paired tumor and adjacent normal tissue samples. Ethical approval for the study protocols was granted by the company’s Ethics Committee under the approval ID: LLS M-15-01.

### 2.3 Immunohistochemistry staining protocol

The protocol for CRC and normal tissue sections was as follows: After paraffin embedding, specimens were subjected to deparaffinization with dimethylbenzene, followed by sequential rehydration through gradient ethanol solutions. Antigen retrieval involved microwave treatment at 95 °C using a sodium citrate buffer. Endogenous peroxidase activity was blocked by incubating tissues in 3% hydrogen peroxide for 10 minutes. To minimize non-specific binding, sections were treated with a blocking solution containing 10% fetal bovine serum for 1 hour. The primary antibody against BTBD19 (1:100 dilution, bioss #bs-8401R) was applied and incubated overnight at 4 °C. Following washing steps, slides were incubated with an HRP-conjugated anti-rabbit secondary antibody in the dark. Immunostaining was visualized using 3,3’-diaminobenzidine, followed by counterstaining with hematoxylin. Tissues were then dehydrated, mounted, and prepared for microscopic examination.

BTBD19 protein expression was evaluated based on two parameters: the proportion of positively stained cells and staining intensity. Positively stained cell percentages were categorized into four groups: category 0 (0–10%), category 1 (10–40%), category 2 (40–70%), and category 3 (>70%). Staining intensity received numerical scores: 1 for weak, 2 for moderate, and 3 for strong signal intensity. The final immunohistochemistry (IHC) score for each sample was calculated by summing the cell positivity score and intensity score, yielding a combined score ranging from 0 to 6. Samples were classified as low-expression (0–3 points) or high-expression (4–6 points). All procedures were executed in adherence to standardized laboratory protocols to ensure methodological consistency and regulatory compliance.

### 2.4 Survival analysis

Survival endpoints, including overall survival (OS), disease-specific survival (DSS), and progression-free interval (PFI), were assessed via the Kaplan-Meier method with log-rank testing. Patients were stratified into low- and high-expression subgroups using the median BTBD19 expression value as the cutoff threshold. Cox proportional hazards regression models were employed to investigate correlations between clinicopathological characteristics and prognostic outcomes, integrating these survival parameters into multivariate analyses to evaluate their independent predictive significance.

### 2.5 Functional enrichment analysis

Differential gene expression analysis between BTBD19 low- and high-expression tissue groups was conducted with significance criteria set as a fold change > 1.5 and false discovery rate (FDR)<0.05 to identify differentially expressed genes (DEGs). The ClusterProfiler package (R v3.6.3) was employed to perform functional enrichment analyses for Gene Ontology (GO) categories—including molecular functions (MF), cellular components (CC), and biological processes (BP)—and Kyoto Encyclopedia of Genes and Genomes (KEGG) pathways, alongside gene set enrichment analysis (GSEA). For GSEA, reference gene sets were derived from the c2.cp.kegg.v2022.1.Hs.symbols.gmt (KEGG pathways) and c5.go.all.v2022.1.Hs.symbols.gmt (GO annotations) databases. Pathway enrichment was evaluated using normalized enrichment scores (NES) and adjusted p-values, with significant enrichment defined as adjusted p<0.05 and FDR<0.25 (11, 12).

### 2.6 Immune infiltration analysis

The ESTIMATE algorithm was applied to calculate immune and stromal scores for CRC samples (13). The GSVA package in R was employed to perform single-sample gene set enrichment analysis (ssGSEA), aiming to explore literature-supported associations between BTBD19 and the hallmark gene signatures of 24 distinct immune cell types. Additionally, CIBERSORT was utilized to investigate the relationship between BTBD19 expression and infiltrating immune cell populations (14, 15). Spearman’s correlation analysis was conducted to assess the association between BTBD19 expression levels and the degrees of immune cell infiltration. Differences in immune cell composition between BTBD19 low- and high-expression groups were evaluated using the Wilcoxon rank-sum test.

### 2.7 Statistical methods

R software (v4.2.1) was employed for statistical analyses of TCGA-derived datasets. To evaluate BTBD19 expression disparities between tumor and normal tissues, Wilcoxon rank-sum tests were applied for independent sample comparisons, while signed-rank tests were utilized for paired sample analyses. Associations between BTBD19 expression and clinicopathological features were assessed using Welch’s one-way ANOVA, followed by Bonferroni *post-hoc* tests for multi-group comparisons (or t-tests for binary group analyses). Pearson’s chi-square test was used to examine correlations between BTBD19 expression and clinical factors, with Fisher’s exact test deployed in cases of small sample sizes to ensure analytical rigor. Prognostic significance of BTBD19 was evaluated via Kaplan-Meier survival curves with log-rank testing. All statistical tests were two-tailed, with significance defined as a threshold of P ≤ 0.05 to maintain consistency in inferential interpretations. For Welch’s one-way ANOVA analyses among multiple groups, Bonferroni *post-hoc* tests were applied for multiple comparison correction to control the family-wise error rate. This correction method adjusts the significance threshold based on the number of comparisons, ensuring that the overall probability of Type I error remains ≤0.05. All statistical results presented for multi-group comparisons have undergone this correction to validate the reliability of the findings.

## 3 Results

### 3.1 Upregulated BTBD19 mRNA and protein levels characterize CRC

**Figure 1:**
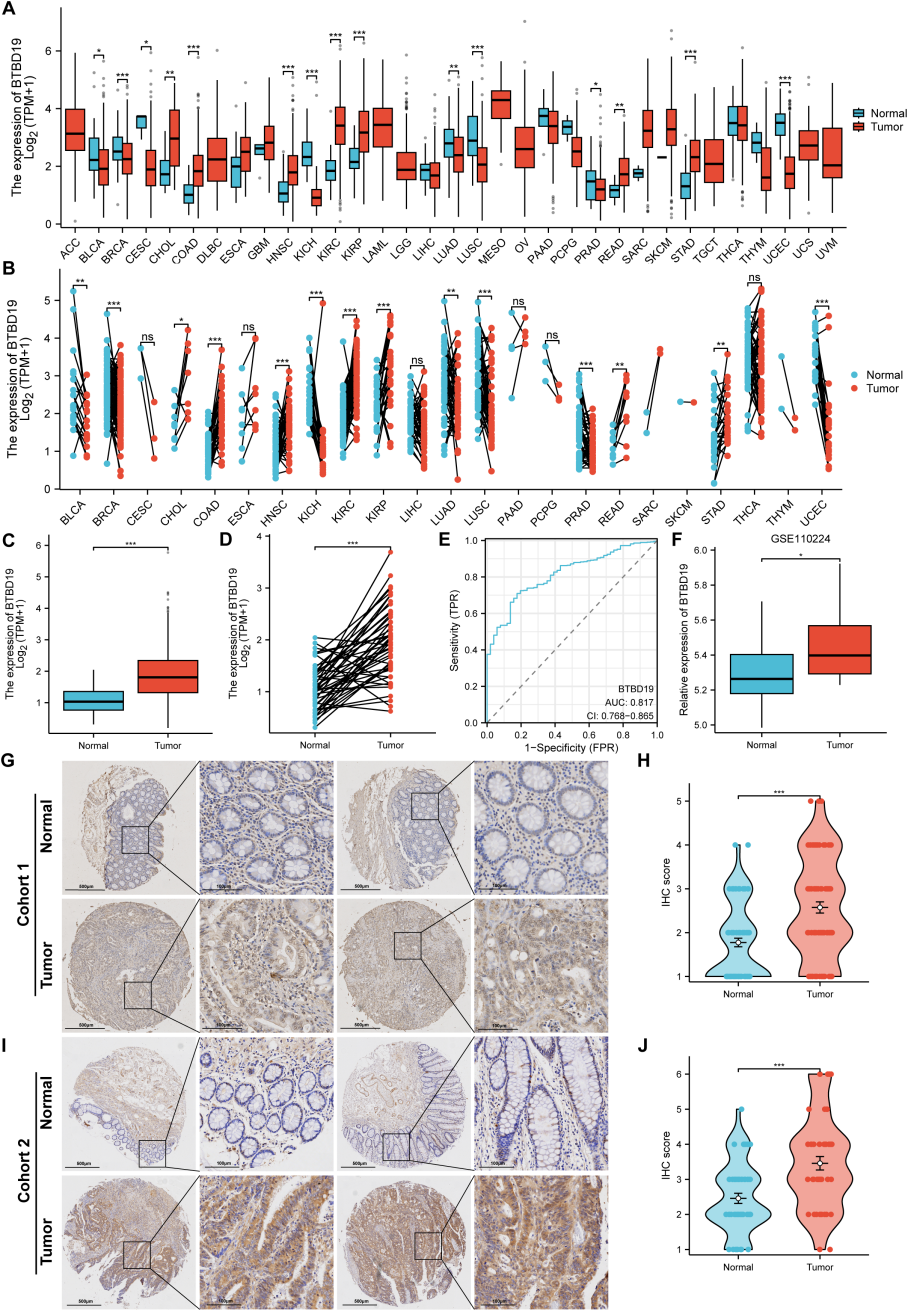
The levels of BTBD19 protein and mRNA expression in pan-cancer and CRC relative to normal samples. **(A, B)** Comparative analysis of BTBD19 mRNA expression in pan-cancer and normal tissues using TCGA. **(C, D)** TCGA-based box-plot and paired-sample analysis showing that BTBD19 mRNA expression was up-regulated in CRC tissues versus normal tissues. **(E)** ROC analysis evaluating the diagnostic value of BTBD19 in CRC. **(F)** Analyses of GEO datasets (GSE110224) revealed up-regulation of BTBD19 mRNA expression in CRC compared with normal samples. **(G, I)** Representative immunohistochemistry images of BTBD19 in normal and CRC tissues **(H, J)** IHC score analysis showing that BTBD19 protein expression was up-regulated in CRC compared with normal samples. (ns denotes no significance, *p<0.05, **p<0.01, ***p<0.001).

The mRNA and protein expression of BTBD19 was explored in pan-cancer and CRC tissues. Data from TCGA were harnessed to contrast BTBD19 mRNA expression across pan-cancer and normal tissues, revealing its expression differed in multiple cancer types (**Figures 1A, B**). Box-plot and paired-sample analyses based on TCGA further showed BTBD19 mRNA expression was notably higher in CRC tissues than in normal ones (**Figures 1C, D**). A ROC analysis was carefully performed to fully evaluate BTBD19’s diagnostic potential in CRC (**Figure 1E**), suggesting it may act as a diagnostic biomarker to partly differentiate tumor states from normal conditions. Analysis of GEO datasets (GSE110224) validated the up-regulation of BTBD19 mRNA in CRC relative to normal samples (**Figure 1F**). IHC staining on clinical CRC specimens was then carried out. IHC score of cohort 1 analysis indicated BTBD19 protein expression was significantly elevated in CRC compared with normal tissues (p<0.001) (**Figure 1H**), with representative immunohistochemistry images visually confirming the expression difference (**Figure 1G**). The IHC results of cohort 2 were similar to those of cohort 1 (**Figures 1I, J**). Collectively, these results demonstrate BTBD19 is prominently overexpressed at both mRNA and protein levels in CRC tissues, hinting at its potential role in CRC tumorigenesis.

### 3.2 Integrative analysis of BTBD19 expression links clinicopathologic features to prognosis in CRC

**Figure 2:**
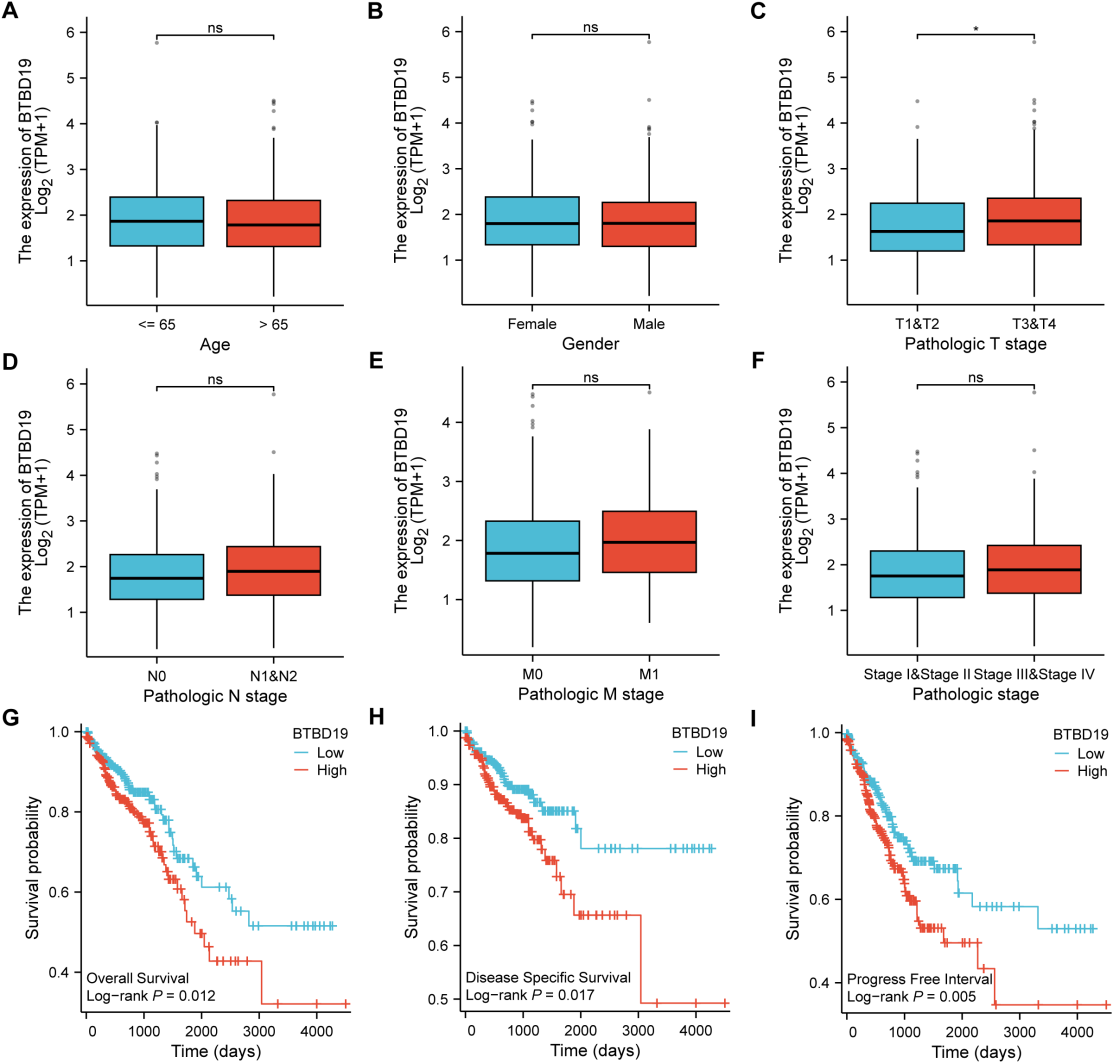
TCGA database-based correlations between BTBD19 expression and clinicopathologic parameters and prognosis in CRC. **(A–F)** Analysis of the association between BTBD19 expression and clinicopathologic parameters in CRC using TCGA data, demonstrating associations with age **(A)**, gender **(B)**, T stage **(C)**, N stage **(D)**, M stage **(E)**, pathologic stage **(F)**. **(G–I)** Kaplan-Meier curves based on TCGA data showing that up-regulated BTBD19 expression was associated with reduced OS event **(G)**, DSS event **(H)**, and PFI event **(I)**. (ns represents no significance, * p<0.05, ** p<0.01, *** p<0.001).

Based on the TCGA database, an analysis was conducted to explore the correlations between BTBD19 expression and clinicopathologic parameters as well as prognosis in CRC. Initially, an exploration into the association between BTBD19 expression and clinicopathologic parameters was carried out. The results showed that BTBD19 expression had no significant connection with age or gender. However, it was notably correlated with T stage (**Figures 2A–F**). Associations between BTBD19 expression and clinicopathological characteristics in 644 CRC patients are summarized in **Table 1**. Compared with those with lower BTDB19 levels, patients with higher expression exhibited more advanced pathological T stage (P = 0.034) and significantly pathological M stage (P = 0.025). Notably, the association between BTBD19 expression and pathological N stage approached statistical significance (P = 0.055). This trend may be influenced by multiple factors, including tumor location-specific biological characteristics, variations in lymphatic vessel density across tumor subtypes, and relatively small sample sizes in certain N-stage subgroups. Subsequently, Kaplan-Meier analysis was performed using TCGA data. This analysis further revealed that when BTBD19 expression was up-regulated, it was associated with a reduction in OS, DSS, and PFI times. Specifically, the log-rank P values were 0.012 for OS, 0.017 for DSS, and 0.005 for PFI (**Figures 2G–I**). These findings strongly imply that high BTBD19 expression might be linked to a poor prognosis in CRC, suggesting that BTBD19 could potentially serve as a valuable biomarker for predicting the outcome of CRC patients.

**Table 1:** The association of BTBD19 expression with clinicopathological features in the TCGA cohort.

Characteristics	Low expression of BTBD19	High expression of BTBD19	P value
n	322	322	
Pathologic T stage, n (%)			0.034
T1-T2	76 (11.9%)	55 (8.6%)	
T3-T4	243 (37.9%)	267 (41.7%)	
Pathologic N stage, n (%)			0.055
N0	196 (30.6%)	172 (26.9%)	
N1-N2	124 (19.4%)	148 (23.1%)	
Pathologic M stage, n (%)			0.025
M0	243 (43.1%)	232 (41.1%)	
M1	34 (6%)	55 (9.8%)	
Pathologic stage, n (%)			0.114
Stage I-Stage II	184 (29.5%)	165 (26.5%)	
Stage III-Stage IV	127 (20.4%)	147 (23.6%)	
Gender, n (%)			0.937
Female	151 (23.4%)	150 (23.3%)	
Male	171 (26.6%)	172 (26.7%)	
Age, n (%)			0.339
<= 65	132 (20.5%)	144 (22.4%)	
> 65	190 (29.5%)	178 (27.6%)	

### 3.3 Biological roles and pathway enrichment analysis of BTBD19 in CRC

**Figure 3:**
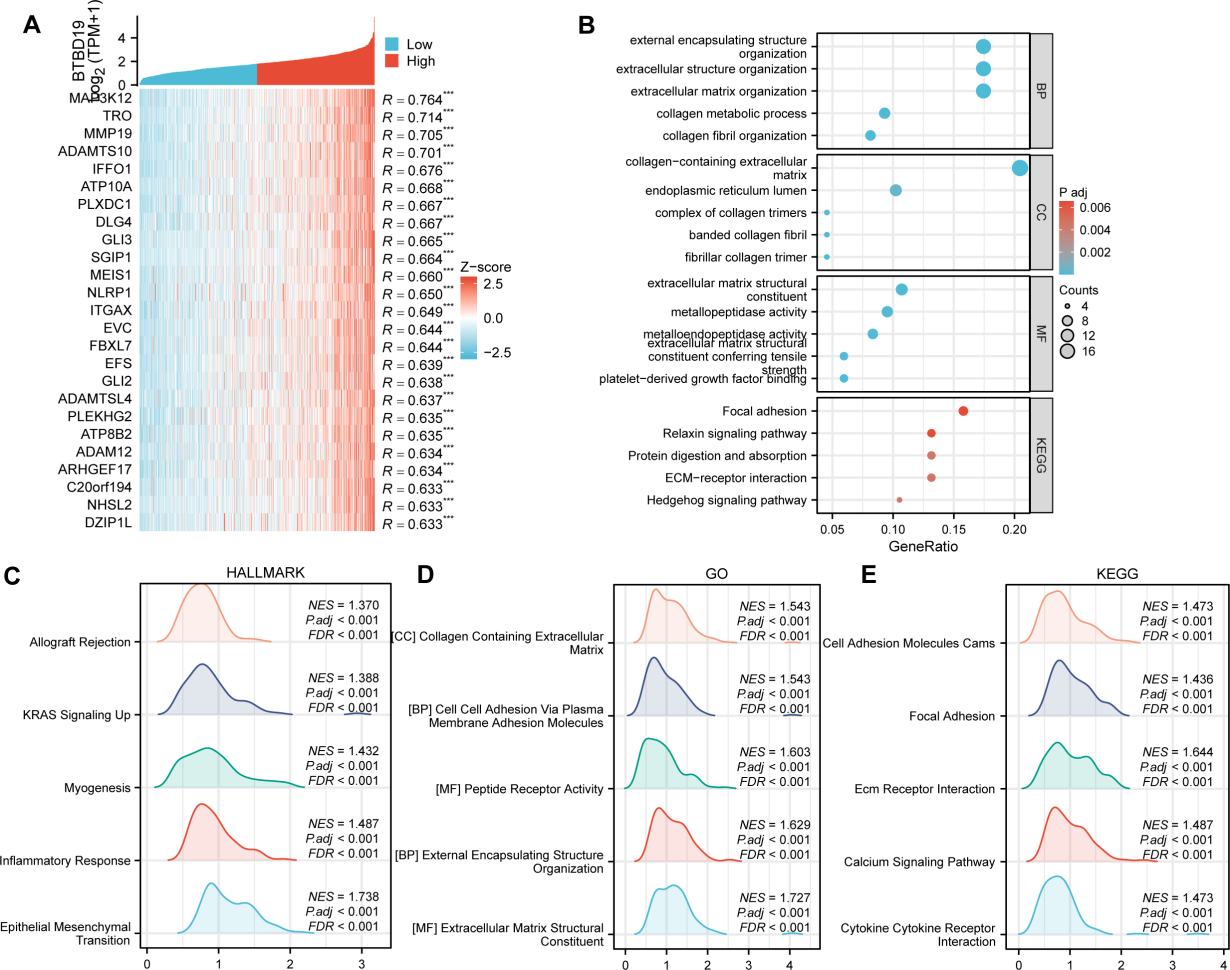
**(A)** Heatmap depicting the top 20 genes in CRC with significant correlation (positive) to BTBD19, where red and blue represent high and low expression-related correlation levels, respectively. **(B)** GO and KEGG Pathway enrichment analyses of co-expressed genes with BTBD19, highlighting significantly enriched biological pathways and functions. **(C–E)** GSEA plots. **(C)** Hallmark gene sets, **(D)** GO-related gene sets, and **(E)** KEGG Pathway-related gene sets, showing pathways significantly correlated with BTBD19 expression in CRC.

To delve deeper into the biological functions of BTBD19 in CRC, a comprehensive analysis of co-expressed genes and enriched functional pathways was undertaken. Initially, the top 20 genes displaying the most significant correlation with BTBD19 expression (**Figure 3A**) were carefully selected and presented in a heatmap, seeking to uncover the molecular mechanisms governing BTBD19-mediated biological processes in CRC. GO and KEGG pathway analyses of co-expressed genes further revealed that BTBD19 is significantly enriched in biological processes such as “external encapsulating structure organization”, “extracellular structure organization”, and “extracellular matrix organization”. These processes are fundamental to shaping the tumor microenvironment, influencing critical oncogenic behaviors like cell adhesion, migration, and invasion—hallmarks of CRC progression. At the cellular component level, enrichment in “collagen-containing extracellular matrix” and “endoplasmic reticulum lumen” implies roles in maintaining structural integrity and intracellular protein homeostasis, both vital for tumor cell survival and proliferation. Molecular functions such as “extracellular matrix structural constituent” and “metallopeptidase activity” further underscore its involvement in ECM remodeling, a process often exploited in cancer to promote tumor growth and metastatic dissemination. KEGG pathways including “Focal adhesion” (implicated in tumor cell motility) and “Relaxin signaling pathway” (linked to angiogenesis and tissue remodeling) were prominently enriched (**Figure 3B**), highlighting BTBD19’s potential impact on CRC progression through microenvironmental modulation.

GSEA (**Figures 3C–E**) further illuminated distinct functional landscapes. Hallmark gene sets (**Figure 3C**) showed enrichment in “Epithelial Mesenchymal Transition (EMT)”, a pivotal process driving metastatic dissemination; “Inflammatory Response”, which nurtures a pro-tumor inflammatory niche; “Myogenesis”, suggesting aberrant muscle-related gene expression that may alter tumor stroma dynamics; “Allograft Rejection”, hinting at immune evasion mechanisms; and “KRAS Signaling Up”, a pathway frequently mutated in CRC to sustain oncogenic signaling. GO-related gene sets (**Figure 3D**) emphasized “Extracellular Matrix Structural Constituent” and “Collagen Containing Extracellular Matrix”, reinforcing BTBD19’s role in ECM-mediated tumor cell interactions. KEGG GSEA (**Figure 3E**) underscored activation of “Ecm Receptor Interaction” (regulating cell-matrix crosstalk), “Calcium Signaling Pathway” (critical for tumor cell survival and proliferation), “Cytokine Receptor Interaction” (modulating tumor-associated inflammation), and “Focal Adhesion” pathways. These collectively regulate processes central to CRC pathogenesis, such as invasive growth and microenvironmental adaptation.

Collectively, these findings provide a multi-dimensional perspective on the functional implications of BTBD19 in CRC, linking it to diverse biological processes, cellular components, molecular functions, and signaling pathways that collectively influence CRC progression. Beyond mere descriptive analysis, these results establish a mechanistic framework: BTBD19 likely impacts CRC through modulating ECM dynamics, tumor-microenvironment interactions, and key oncogenic signaling pathways. Such insights not only advance our understanding of BTBD19’s role in colorectal oncogenesis but also highlight actionable pathways for therapeutic intervention, guiding future studies to explore BTBD19 as a potential biomarker or therapeutic target in CRC.

### 3.4 Association of BTBD19 expression with immune cell infiltration in CRC

**Figure 4:**
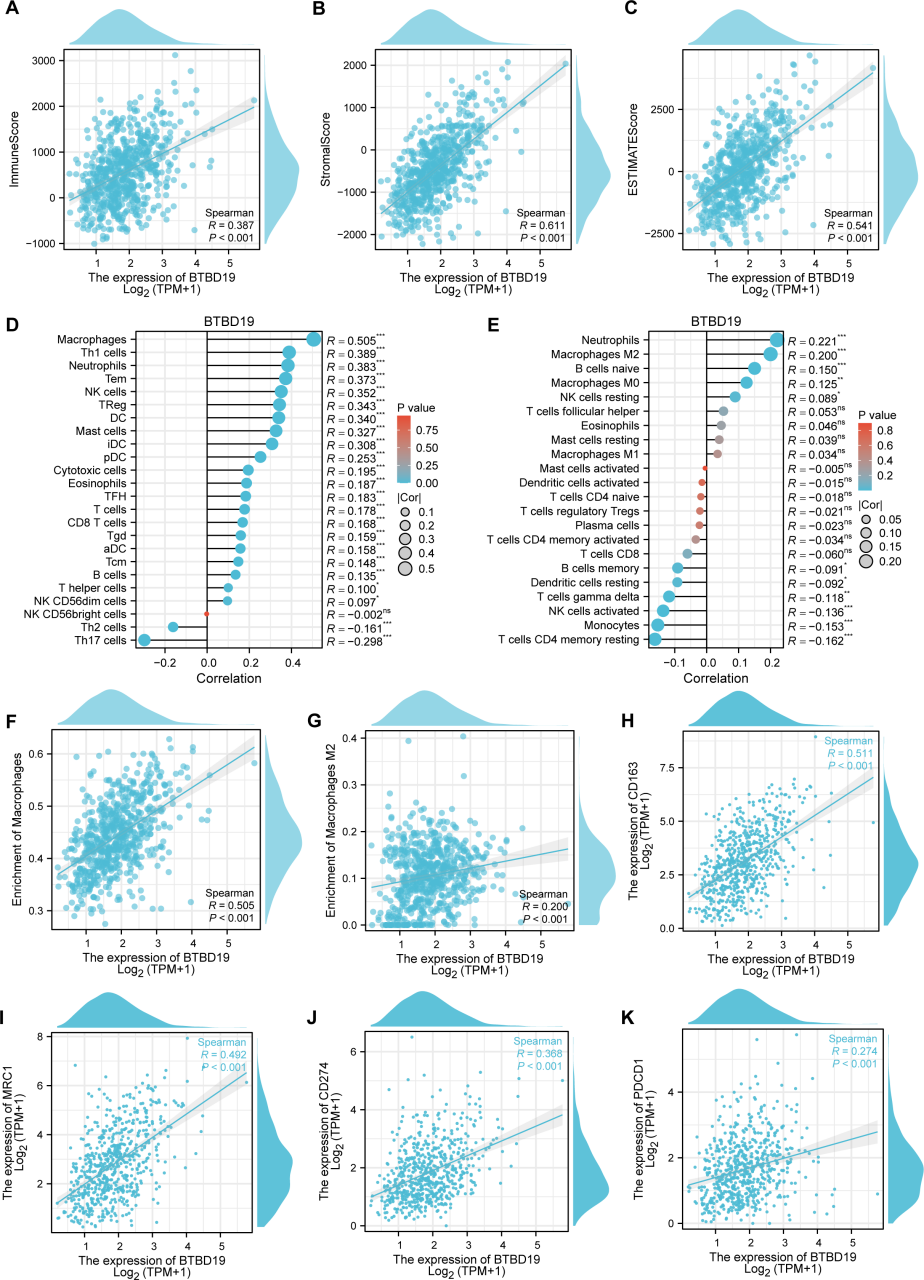
The correlation between BTBD19 and immune cells in CRC. **(A–C)** The ESTIMATE algorithm was used to analyze the association of BTBD19 expression with the ESTIMATE score, stromal score, and immune score. **(D, E)** The relationship between BTBD19 gene expression and immune cell infiltration in CRC was explored via ssGSEA. **(F)** Correlation analysis between BTBD19 and macrophages. **(G)** Correlation analysis between BTBD19 and M2 macrophages. **(H, I)** Associations between BTBD19 expression levels and macrophage markers (CD163, MRC1). **(J, K)** Associations between BTBD19 expression levels and immune checkpoints (CD274, PDCD1) (ns represents no significance, * p<0.05,** p<0.01, *** p<0.001).

To investigate the association between BTBD19 and immune cells in CRC, the ESTIMATE algorithm was applied (**Figures 4A–C**). Findings uncovered positive correlations between BTBD19 expression and the ESTIMATE score (R = 0.541, P<0.001), stromal score (R = 0.611, P<0.001), as well as the immune score (R = 0.387, P<0.001), suggesting its linkage to tumor microenvironment components. Through ssGSEA analysis, BTBD19 was identified to significantly correlate with multiple immune cell types. Notably, BTBD19 expression demonstrated a robust correlation with macrophage infiltration (R = 0.505, P<0.001), particularly with M2 macrophages (R = 0.200, P<0.001) via CIBERSORT-key constituents of the TME (**Figures 4D, E**). M2 macrophages are known to mediate diverse pro-tumor mechanisms, including angiogenesis, ECM remodeling, cancer cell proliferation, metastasis, immunosuppressive signaling, chemotherapeutic resistance, and reduced sensitivity to immune checkpoint blockade therapy. These relationships imply that BTBD19 may engage with M2 macrophage-associated pathways to modulate CRC progression and tumor-immune interactions. Further exploration (**Figure 4F**) displayed a positive association between BTBD19 and macrophages (R = 0.505, P<0.001), while **Figure 4G** illustrated its correlation with M2 macrophages (R = 0.200, P<0.001). Links between BTBD19 expression and macrophage markers CD163 (R = 0.511, P<0.001) and MRC1 (R = 0.492, P<0.001) were also detected (**Figures 4H, I**). Furthermore, BTBD19 expression correlated with other immune checkpoints CD274 (R = 0.368, P<0.001) and PDCD1 (R = 0.274, P<0.001) (**Figures 4J, K**), further highlighting its role in immune-cell-related processes. Altogether, these results indicate that BTBD19 may exert influence on immune-cell-related processes in CRC, potentially affecting tumor-immune crosstalk.

### 3.5 Association of BTBD19 expression with cytokine and immune-related factors in CRC

**Figure 5:**
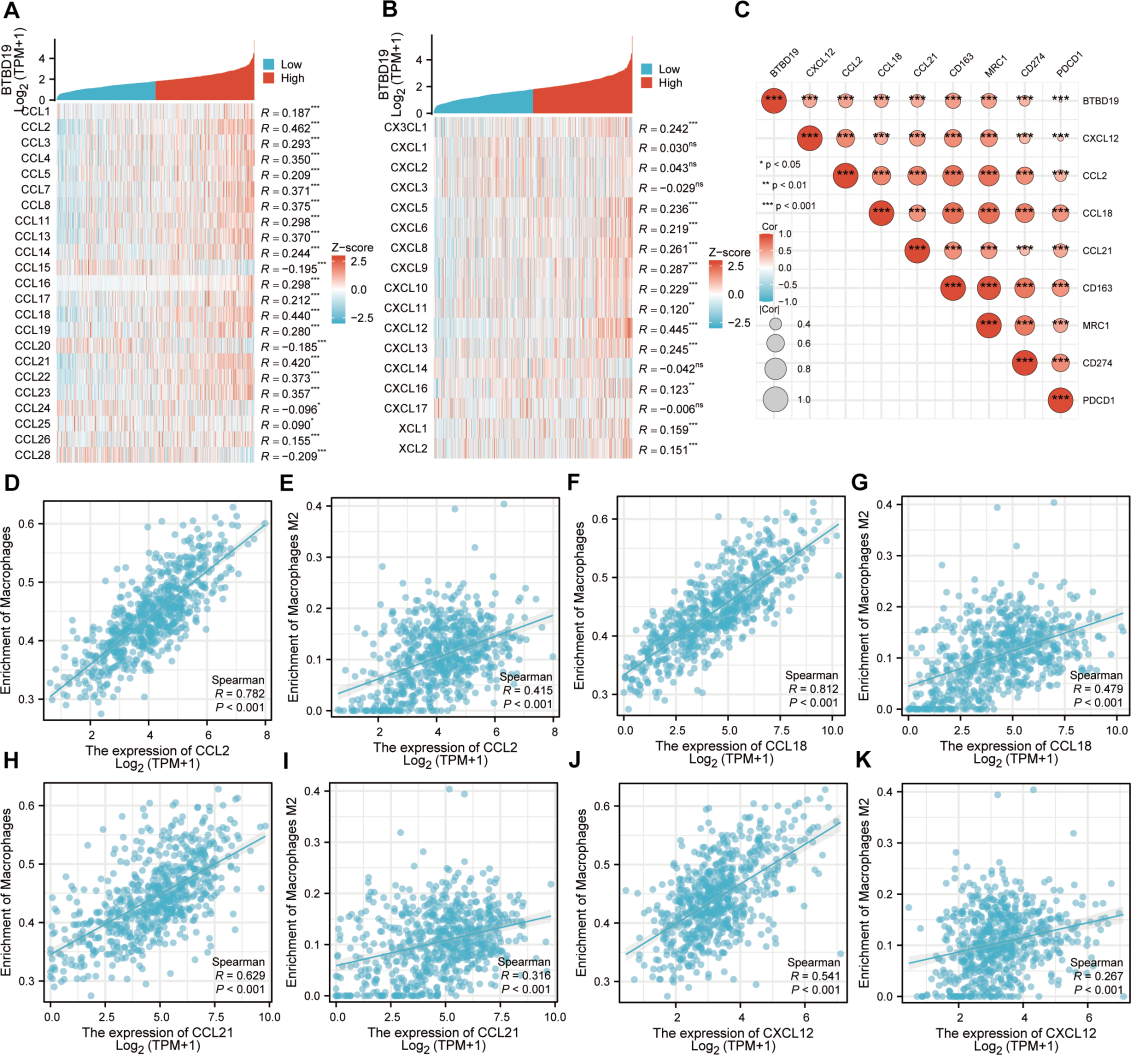
**(A, B)** Correlation analysis of BTBD19 gene expression with cytokines (CCL, CXCL) in CRC. **(C)** The associations between the expression levels of BTBD19, Strong correlation cytokines (CXCL12, CCL2, CCL18, CCL21), macrophage markers (CD163, MRC1) and immune checkpoints (CD274, PDCD1). **(D, E)** Correlation analysis between CCL2 expression and Macrophages cell infiltration in CRC. **(F, G)** Correlation analysis between CCL18 expression and Macrophages cell infiltration in CRC. **(H, I)** Correlation analysis between CCL21 expression and Macrophages cell infiltration in CRC. **(J, K)** Correlation analysis between CCL12 expression and Macrophages cell infiltration in CRC.

Given the potential of cancer cells to modulate immune cell polarization via chemokines and their receptors, this study investigated the relationship between BTBD19 expression and chemokine/receptor profiles sourced from the TISIDB database (**Figures 5A, B**). In general, the highest correlation was observed for CCL2 (R = 0.462), CCL18 (R = 0.440), CCL21 (R = 0.420) and CXCL12 (R = 0.445) among cytokines. We thus further investigated the relationship between the above cytokines and immune cells, and the results were similar to those of the BTBD19 gene itself (**Figures 5D–K**). A correlation network was presented, demonstrating significant positive associations between BTBD19, cytokines (CXCL12, CCL2, CCL18, CCL21), macrophage markers (CD163, MRC1) and immune checkpoints (CD274, PDCD1) (**Figure 5C**).

## 4 Discussion

CRC is a biologically heterogeneous malignancy characterized by disrupted cytokine signaling networks (16). These networks interfere with numerous cellular pathways, propelling tumor initiation, progression, and the emergence of aggressive phenotypic characteristics (17). Elucidating the intricate molecular mechanisms underlying CRC development is essential for advancing early detection techniques, refining treatment protocols, and strengthening the ability to regulate disease progression (18). A vital subsequent step entails identifying novel biological markers linked to immune cell infiltration patterns and disentangling the fundamental molecular pathways that govern responses to immunotherapeutic interventions (19, 20). Such an approach aims to enhance our understanding of CRC diversity and foster the development of tailored strategies to elevate clinical outcomes (21). Our study unveils BTBD19 as a novel oncogenic driver in CRC, linking its overexpression to aggressive clinicopathological features, immune microenvironment remodeling, and adverse patient outcomes. This work extends the understanding of BTBD family proteins in cancer, particularly in the context of colorectal carcinogenesis, where BTBD19 emerges as a previously uncharacterized regulator.

Our study demonstrates that BTBD19 is significantly upregulated in CRC at both mRNA and protein levels, consistent across TCGA, GEO datasets, and clinical IHC samples. High BTBD19 expression correlated with aggressive clinicopathologic features and poor survival outcomes (OS, DSS, PFI), establishing it as a potential prognostic indicator. This aligns with prior studies on other BTBD family members that modulate tumor progression through diverse mechanisms.

Functional enrichment analyses revealed BTBD19’s involvement in critical biological processes, including ECM organization, focal adhesion, and EMT. ECM remodeling is a hallmark of cancer progression, enabling tumor cell invasion, metastasis, and angiogenesis (22). The enrichment of “collagen-containing ECM” and “metallopeptidase activity” suggests BTBD19 may facilitate CRC cell motility and basement membrane degradation, processes essential for metastatic dissemination.

GSEA further linked BTBD19 to activated KRAS signaling and EMT, both of which are frequently dysregulated in CRC and associated with poor prognosis. BTBD19 expression is associated with enhanced tumor cell invasiveness, which may contribute to CRC progression. Using the ESTIMATE algorithm, positive associations were observed between BTBD19 expression and stromal scores (R = 0.611, P<0.001), as well as immune scores (R = 0.387, P<0.001), underscoring its role in sculpting the structural and immune components of the TME. A pivotal discovery was the robust link between BTBD19 levels and macrophage infiltration, particularly with M2 macrophages-key drivers of tumor-promoting inflammation. Analyses using ssGSEA and CIBERSORT revealed a strong correlation with total macrophage abundance (R = 0.505, P<0.001), especially M2 macrophages (R = 0.200, P<0.001). This association was further validated by direct correlations with macrophage-specific markers CD163 (R = 0.511, P<0.001) and MRC1 (R = 0.492, P<0.001). M2 macrophages are recognized for releasing growth factors and matrix-degrading enzymes, thereby facilitating angiogenesis, ECM remodeling, and cancer cell invasion. Furthermore, they suppress antitumor immune responses through the production of IL-10 and TGF-β, which inhibit T-cell activation and promote regulatory T-cell recruitment (23, 24). The enrichment of BTBD19 in M2-associated pathways implies that it may drive macrophage polarization toward an immunosuppressive phenotype, establishing a microenvironment conducive to tumor progression and resistance to immunotherapeutic interventions. BTBD19 expression was also significantly associated with immune checkpoint molecules PD-L1 (CD274, R = 0.368, P<0.001) and PD-1 (PDCD1, R = 0.274, P<0.001), key regulators of T-cell exhaustion. This association highlights a potential mechanistic link between BTBD19 and tumor immune evasion: upregulated PD-L1/PD-1 signaling within the TME dampens cytotoxic T-cell activity, thereby enabling tumors to circumvent immune surveillance. Notably, this interplay may underlie the observed poor prognosis in BTBD19-high CRC patients, as PD-L1/PD-1 axis activation is strongly associated with immunosuppressive tumor microenvironments (25). Clinically, these findings suggest that BTBD19 expression could serve as a predictive biomarker for response to immune checkpoint blockade, with high BTBD19 levels potentially indicating reduced sensitivity to PD-1/PD-L1 inhibitors-a critical consideration for personalized treatment strategies in CRC. The correlation analysis extended to cytokine-receptor networks revealed that BTBD19 was strongly linked to CCL2, CCL18, CCL21, and CXCL12—key chemokines central to immune cell trafficking and TME organization. These cytokines act as molecular cues for recruiting immune cells, particularly monocytes and macrophages, into the tumor niche. For instance, CCL2 (MCP-1) and CXCL12 (SDF-1α) are well-documented drivers of monocyte chemotaxis, promoting their differentiation into M2-like tumor-associated macrophages (TAMs) that foster angiogenesis and immunosuppression (26). The positive correlation between BTBD19 and these chemokines suggests that BTBD19 may upregulate chemokine expression to recruit M2 macrophages, while M2 TAMs reciprocally secrete additional chemokines and growth factors to sustain BTBD19-mediated tumor progression. This interplay is further validated by the correlation network (**Figure 5C**), which demonstrates co-expression between BTBD19, M2 markers, and chemokines. Clinically, this network highlights BTBD19 as a potential upstream regulator of chemokine signaling, offering a rationale for combinatorial therapies targeting both BTBD19 and its associated cytokine axes to disrupt TME homeostasis. The correlation analysis extended to cytokine-receptor networks revealed that BTBD19 was strongly linked to the CCL2, CCL18, CCL21, CXCL12, which is central to immune cell trafficking and TME organization. CXCL12, a chemokine highly correlated with BTBD19 (R = 0.445), promotes macrophage recruitment and M2 polarization. BTBD19 may upregulate CXCL12 to recruit M2 macrophages, and together they may reinforce immunosuppressive signaling and ECM remodeling, thereby promoting CRC progression. These interactions likely drive a feedforward loop where BTBD19 upregulates chemokine signaling, attracting immunosuppressive cells while repelling cytotoxic T cells, thereby fostering a pro-tumor microenvironment. In summary, BTBD19 emerges as a pivotal node in TME biology, integrating ECM remodeling, immune cell recruitment, and checkpoint signaling to promote a pro-tumor microenvironment. These findings deepen our understanding of CRC-immune interactions and highlight BTBD19 as a candidate for stratified therapy based on TME characteristics. Moreover, the association with CCL18 and CCL21-known for recruiting regulatory T cells (Tregs) and dendritic cells-implies BTBD19 may also modulate adaptive immune responses (27, 28). These findings collectively position BTBD19 as a pivotal node in cytokine-immune cell crosstalk, underscoring its multifaceted role in shaping a pro-tumorigenic microenvironment. Future studies exploring BTBD19-chemokine interactions could uncover novel strategies to rewire the TME for enhanced immunotherapy efficacy in CRC. Beyond the prognostic value of BTBD19, exploring its interplay with emerging therapeutic strategies could enhance clinical utility. Recent advancements highlight the potential of repurposing drugs such as GLP-1-based therapies or proteasome-targeting agents, alongside natural compounds like prodigiosin or hinokitiol, which exhibit prophylactic effects and immuno-modulatory properties in cancer contexts (29–32). Investigating whether BTBD19 expression correlates with responsiveness to these interventions could inform personalized treatment regimens, though further preclinical studies are warranted to validate such associations.

While this study provides a comprehensive bioinformatics and immunohistochemical analysis, several limitations warrant mention. First, the lack of in vitro/in vivo functional experiments (e.g., BTBD19 knockdown/overexpression cell assays, animal models of metastasis) precludes direct verification of its mechanistic role in CRC progression. Second, upstream regulatory mechanisms of BTBD19, such as transcription factors or epigenetic modifications, remain unexplored, limiting our understanding of its expression regulation. Third, the specific downstream targets and pathways linking BTBD19 to ECM remodeling and EMT require further validation (e.g., interactions with collagen, MMPs, or EMT markers). Notably, the in silico predictions of BTBD19-associated immune cell infiltration (e.g., enrichment of M2 macrophages) provide compelling working hypotheses, but their biological validity requires further validation using orthogonal experimental approaches. In our future studies, we plan to verify these correlations using techniques such as multiplex immunohistochemistry (IHC) or flow cytometry on independent CRC patient samples, which will help confirm the in situ distribution and functional relevance of immune cells associated with BTBD19 expression. Future studies addressing these aspects will strengthen the translational potential of our findings. BTBD19’s upstream regulators and downstream effectors, as well as its potential as a prognostic biomarker through small-molecule inhibitors or immune-modulatory strategies.

## 5 Conclusions

In summary, this study identifies BTBD19 as a novel contributor to CRC progression, linking its overexpression to aggressive clinicopathological features, ECM remodeling, and immune microenvironment dysregulation. BTBD19’s dual roles in promoting cell proliferation and shaping a pro-tumor immune landscape highlight its potential as a prognostic biomarker for CRC. Further mechanistic investigations and translational research are essential to unlock its clinical utility, particularly in developing personalized strategies that integrate BTBD19 status with immune checkpoint profiling.

## Data Availability

RNA-seq datasets with corresponding clinical information from colorectal cancer (CRC) tumors and adjacent normal tissue samples were acquired from The Cancer Genome Atlas (TCGA, https://cancergenome.nih.gov/), a leading public resource for cancer genomics research. Additionally, the GSE110224 dataset utilized in this study was extracted from the Gene Expression Omnibus (GEO, http://www.ncbi.nlm.nih.gov/geo), a broadly accessible functional genomics repository that facilitates MIAME-standard data submissions.
